# Zero-power infrared switch with two-phase microfluidic flow and a 2D material thermal isolation layer

**DOI:** 10.1038/s41378-024-00761-x

**Published:** 2024-09-02

**Authors:** Zekun Zhang, Peng Li, Yixuan Zou

**Affiliations:** 1 Department of Precision Instruments, Tsinghua University, 100084 Beijing, China; 2 School of Mechanical Science and Engineering, Huazhong University of Science and Technology, 430074 Wuhan, China

**Keywords:** Nanoscience and technology, Engineering

## Abstract

Wireless sensor nodes (WSNs) play an important role in many fields, including environmental monitoring. However, unattended WSNs face challenges in consuming power continuously even in the absence of useful information, which makes energy supply the bottleneck of WSNs. Here, we realized zero-power infrared switches, which consist of a metasurface and two-phase microfluidic flow. The metasurface can recognize the infrared signal from the target and convert it into heat, which triggers the two-phase microfluidic flow switch. As the target is not present, the switch is turned off. The graphene/MoS_2_/graphene 2D material heterostructure (thickness <2 nm) demonstrates an exceptionally high thermal resistance of 4.2 K/W due to strong phonon scattering and reduces the heat flow from the metasurface to the supporting substrate, significantly increasing the device sensitivity (the displacement of the two-phase microfluidic flow increases from ~1500 to ~3000 µm). The infrared switch with a pair of symmetric two-phase microfluidic flows can avoid spurious triggering resulting from environmental temperature changes. We realized WSNs with near-zero standby power consumption by integrating the infrared switch, sensors, and wireless communication module. When the target infrared signal appears, the WSNs are woken and show superb visual/auditory sensing performance. This work provides a novel approach for greatly lengthening the lifespan of unattended WSNs.

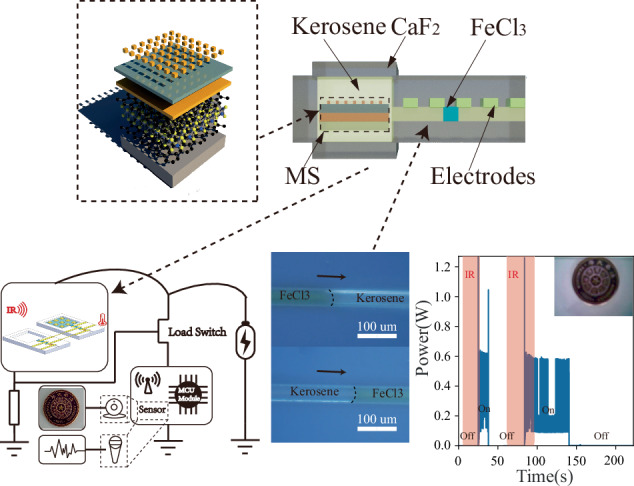

## Introduction

With the development of the Internet of Things (IoT), a multitude of wireless sensor nodes (WSNs) have been deployed, which play crucial roles in industrial automation, environmental monitoring, and many other fields^1–4^. However, the application of unattended WSNs is currently constrained by the energy supply^5^. In scenarios such as forest fires or gas leaks^6,7^, which are infrequent but critical, traditional WSNs powered by batteries face challenges of continuous power consumption even in the absence of useful information^8,9^. Frequently charging or replacing batteries largely reduces the significance of unattended WSNs.

The integration of WSNs with triboelectric or piezoelectric nanogenerators presents a promising avenue for prolonging the lifespan of sensor nodes through the provision of supplementary power sources^10–14^. Nevertheless, the electrical power derived from the current energy harvesting technique tends to be limited and unstable, which makes ensuring the timely functioning of WSNs challenging. Another method of extending the lifespan of sensor nodes is the deployment strategy along with algorithmic adjustments, which can effectively reduce the overall power consumption of large-scale WSN networks^15,16^, but they are unable to completely eliminate substantial standby power consumption.

Employing devices that harness the signal/energy from the target to awake sensor nodes is a promising strategy that can largely reduce energy consumption in standby mode^8,17,18^. However, an electrical circuit is needed to derive the wake-up signal. Although low-power electrical circuits can be used, standby power consumption cannot be avoided. Therefore, devices that can generate wake-up signals without the need for electrical circuits are highly desirable. Truong et al. fabricated a MEMS (microelectromechanical system) switch with a 5 nm gap between the microcantilever and the substrate^19^. As target gas molecules were captured by the molecular chains decorated on the microcantilever and substrate surfaces, the switch was turned on. However, the extremely small gap (5 nm) between the microcantilever and substrate poses challenges to device manufacturing and stability. Qian et al. developed a near-zero-standby-power infrared switch by employing a metasurface to convert infrared energy into heat and actuated a microcantilever beam to bend and connect the metal pad^20^. Since the infrared energy emitted from the target is extremely low, elongated microcantilever beams and extremely small gaps are needed. As such, device fabrication is extremely challenging and has the problem of easy spurious triggering by vibration and electrostatic attraction. Moreover, the device lacked self-holding ability, indicating that it rapidly turned off once the target infrared radiation disappeared, which did not provide sufficient time for WSNs to complete data acquisition and processing. Therefore, there is an urgent need for easily fabricated, reliable, zero-standby-power WSNs with self-holding ability.

In this work, we developed an easily fabricated, reliable, zero-power infrared switch by integrating a metasurface (MS), two-phase microfluidic flow, and a 2D material heterostructure thermal isolation layer. The metasurface can recognize the infrared signals emitted from the target and convert them into heat, which turns on the two-phase microfluidic flow switch. The two-phase microfluidic flow switch is slowly turned off as the target infrared signal disappears, demonstrating self-holding ability. On the basis of this technique, we realized near-zero standby power WSNs.

## Experiment

### Fabrication of the MS with a 2D material thermal isolation layer

The MS has a metal‒insulator‒metal (MIM) sandwich structure on a graphene/MoS_2_/graphene heterostructure. Chemical vapor deposition (CVD)-grown monolayer graphene/MoS_2_/graphene was transferred onto a 200 µm-thick glass substrate as a thermal isolation layer. Then, 5 nm Cr/40 nm Au/5 nm Cr (reflection layer) was deposited on the thermal isolation layer and patterned via photolithography, electron-beam evaporation, and a metal lift-off process. A 300-nm-thick silicon nitride film (dielectric layer) was subsequently deposited via plasma-enhanced chemical vapor deposition (PECVD) on the reflection layer. The metal arrays were fabricated on top of the dielectric layer via electron-beam lithography, evaporation, and metal lift-off.

### Fabrication of the two-phase microfluidic switch

A glass substrate with a liquid reservoir and microchannel (channel width <40 µm) was fabricated with a laser cutting machine. The electrodes on the glass substrate were derived via photolithography, electron-beam evaporation, and metal lift-off. The MA was placed into the reservoir. Then, the glasses were bonded together with a UV-curable adhesive. The liquid reservoir and part of the microchannel were filled with kerosene as an insulating liquid via the vacuum-assisted method. A trace amount of conductive FeCl_3_ solution (~0.1 nL) was filled into the microchannel and formed a two-phase microfluidic flow with kerosene.

### Device measurements

We employed a 170-mW blackbody light source, CaF_2_ glasses, ZnS lenses, and various filters to generate infrared light with different wavelengths and irradiate the infrared switch. A digital multimeter (Keysight 34470) was used to measure the resistance/current of the two-phase microfluidic switch in the infrared switch. A digital power meter was used to measure the power consumption of the unattended WSNs.

## Results and discussion

### Device structure and working mechanism

**Fig. 1:**
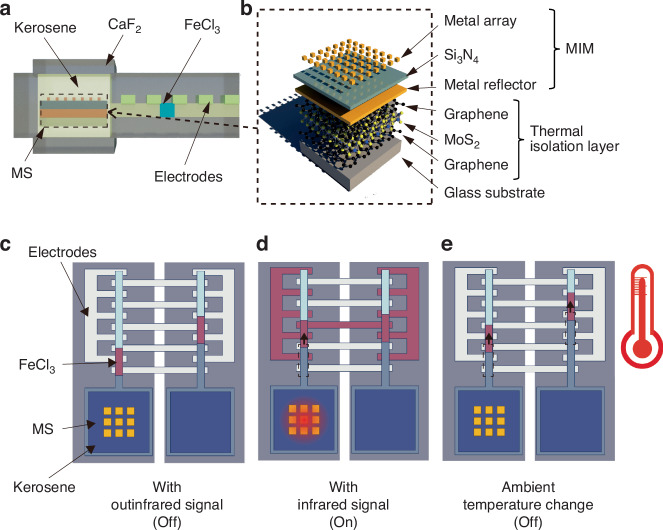
Infrared switch. **a** Cross-sectional view of the infrared switch with the MS and a two-phase microfluidic switch. **b** Schematic view of the MS with a graphene/MoS_2_/graphene 2D material heterostructure thermal isolation layer. **c** Infrared switch in the ‘off’ state without a target infrared signal (top view). **d** Infrared switch in the ‘on’ state with a target infrared signal. **e** The infrared switch can avoid spurious triggering resulting from environmental temperature changes

As shown in Fig. 1a, b, the infrared switch consists of two two-phase microfluidic switches. Each two-phase microfluidic switch comprises a microchannel with electrode arrays and a reservoir. The reservoir and part of the channel are filled with kerosene. Kerosene is chosen because it is transparent to infrared signals. Insulating kerosene and conductive FeCl_3_ solution (two immiscible solutions) form a two-phase microfluidic flow in the microchannel. The MS with a 2D material thermal isolation layer is placed in the reservoir and completely immersed in kerosene (only 1 microfluidic switch is integrated with the MS). The infrared signal passes through an infrared-transparent CaF_2_ glass and kerosene and irradiates the MS, which converts the infrared signal into heat. The heat results in kerosene expansion in the liquid reservoir, thereby pushing the trace FeCl_3_ solution forward in the microchannel. When the infrared energy absorbed by the MS surpasses a certain value, the trace FeCl_3_ solution is connected to the proper pair of metal electrodes, and the switch is turned on, as shown in Fig. 1d. Under ambient temperature variation, the FeCl_3_ solutions in both microchannels move simultaneously without activating the infrared switch, indicating that spurious triggering resulting from environmental temperature changes can be avoided.

### Metasurface

**Fig. 2:**
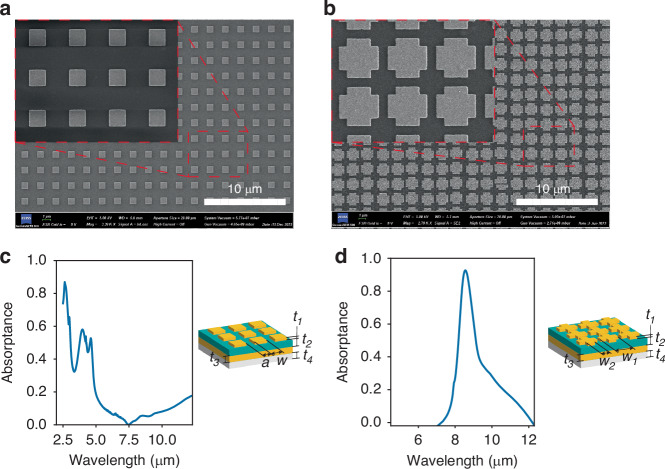
Metasurface. **a** SEM image of the MS with an absorption wavelength of 2–5 μm. **b** SEM image of the MS with an absorption wavelength of 9 μm. **c**, **d** Absorption spectra of the MAs characterized by FTIR

The MS can recognize the infrared signals emitted from the target and convert the signals into heat. Compared with traditional infrared absorbing materials, the MS is much smaller in size and has better absorptivity. It also has excellent wavelength selectivity^21–24^. The MS has a metal-insulator-metal (MIM) sandwich structure. The transmittance of the MS is negligible owing to the reflection layer. The geometrical sizes of the top metal arrays are crucial for the absorptivity and absorption wavelength. The impedance of the MS should match the free space impedance:

1 $${Z}_{0}=\sqrt{\mu (\omega )/\epsilon (\omega )}$$

where Z_0_ is the characteristic impedance of free space, *μ*(*ω*) is the magnetic permeability, *ϵ*(*ω*) is the permittivity, and *ω* is the frequency. An infrared signal with a frequency matching the absorption spectrum of the MS will be absorbed. To detect events such as forest fires (infrared radiation wavelengths of 2–5 µm^25–27^) and the appearance of humans (infrared radiation wavelengths of 9 µm^28^), we designed two types of MSs with different absorption spectra (wavelengths of 2–5 µm and 9 µm) via the finite-difference time-domain (FDTD) method. The important geometry parameters are shown in Tables 1 and 2. The morphologies of the fabricated MSs were characterized by scanning electron microscopy (SEM), as shown in Fig. 2a, b. Fourier transform infrared spectroscopy (FTIR) was employed to characterize the absorption characteristics of the MSs, which demonstrated exceptional infrared absorptivity (≥85%) and excellent frequency selectivity (Fig. 2c, d).

**Table 1:** Geometric parameters of the MS with an absorption wavelength of 2–5 μm

Parameters	Description	Size (μm)
a	Spacing between metal antennas	1.13
w	Square side length	0.77
t_1_	Metal antenna thickness	0.05
t_2_	Si_3_N_4_ thickness	0.3
t_3_	Bottom metal layer thickness	0.05
t_4_	Glass substrate thickness	200

**Table 2:** Geometric parameters of the MS with an absorption wavelength of 9 μm

Parameters	Description	Size (μm)
a	Spacing between metal antennas	2.2
w_1_	Cross length	1.83
w_2_	Cross width	1.1
t_1_	Metal antenna thickness	0.05
t_2_	Si_3_N_4_ thickness	0.3
t_3_	Bottom metal layer thickness	0.05
t_4_	Glass substrate thickness	200

### Two-phase microfluidic flow

**Fig. 3:**
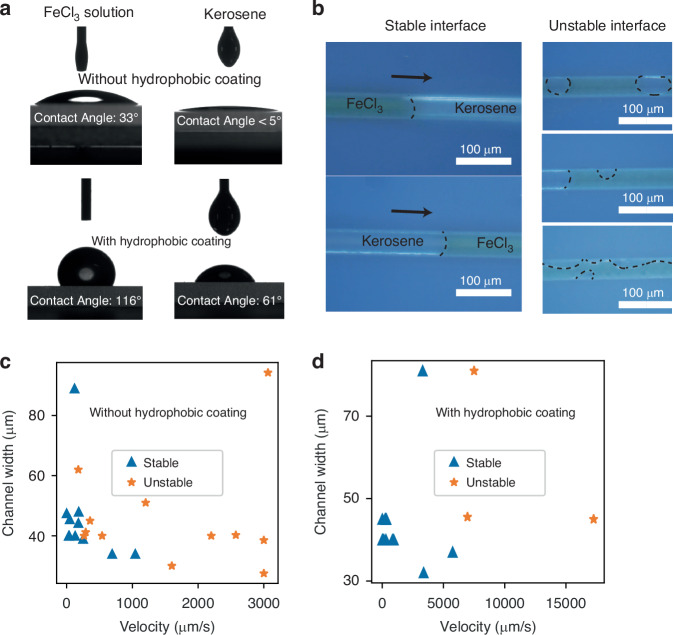
Stability of two-phase microfluidics. **a** Contact angle between FeCl_3_ solution/kerosene and glass with/without a hydrophobic coating. **b** Optical images of stable and unstable two-phase microfluidics in the microchannels. **c** Stability of two-phase microfluidics without a hydrophobic coating. **d** Stability of two-phase microfluidics with a hydrophobic coating

**Fig. 4:**
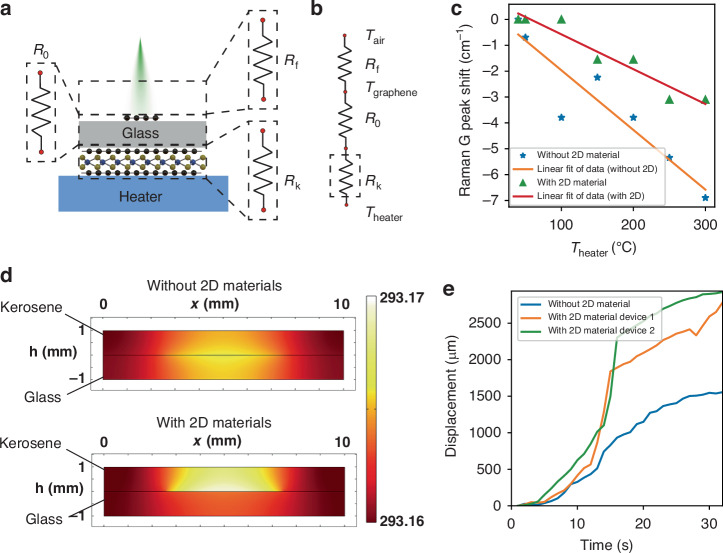
2D material heterostructure thermal isolation layer. **a** Schematic of the thermal resistance measurement experimental setup. **b** Thermal resistance model. *R*_*k*_ is the thermal resistance of the 2D material heterostructure. **c** Raman G peak shift of the top graphene flake under different *T*_*heater*_ with/without *R*_*k*_. Temperature variation of the top graphene flake *∆T*_*graphene*_ can be derived from the Raman G peak shift. **d** Simulation results of the cross-sectional temperature distribution with/without a 2D material heterostructure layer. **e** Experimental results of the displacement of two-phase microfluidics heated by the MS with/without a 2D material heterostructure

To realize a reliable switch, it is essential to keep the two-phase microfluidic interface stable and avoid mixing kerosene and the FeCl_3_ solution during switching. We first investigated the impact of channel surface wettability on the two-phase microfluidic flow. Kerosene has a high affinity for glass surfaces, so it can easily form a wetting layer and envelop the FeCl_3_ solution. To address this problem, we coated the microchannels with silicone polymer as a hydrophobic layer. As a result, the contact angle of kerosene on the glass surface increased from <5 to 61°, and the contact angle of the FeCl_3_ solution increased from 33 to 116° (Fig. 3a). Figure 4b shows stable and unstable kerosene/FeCl_3_ two-phase microfluidic flows. Devices with hydrophobic coatings are more likely to remain stable. For unstable microfluidics, boundary residuals^29^ and the envelopment of aqueous solutions by oil wetting^30^ were observed (Fig. 3b). The impacts of the channel width and flow rate on the properties of the two-phase microfluidic flow were also systematically investigated. Our experimental results indicate that a lower fluid velocity and smaller channel width are essential for realizing stable two-phase microfluidic flow (Fig. 3c, d). Additionally, without a hydrophobic coating, the stable two-phase microfluidic flow can only move at a speed of 1000 µm/s, whereas with a hydrophobic coating, the speed can reach 5000 µm/s. The hydrophobic coating allows the two-phase microfluidic flow to remain stable at relatively high velocities in the microchannel. Reliable two-phase microfluidic switches were realized on the basis of this study. This approach is important for the fundamental research and application of two-phase microfluidics.

### 2D material thermal isolation layer

Kerosene in the reservoir absorbs the heat generated from the MS, resulting in volume expansion ΔV, which can be expressed as:

2 $$\Delta {\rm{V}}=\frac{{\rm{\alpha }}}{{\rm{C}}{\rm{\rho }}}Q$$

where α is the thermal expansion coefficient of kerosene, *C* is the heat capacity, ρ is the density of kerosene, and *Q* is the heat absorbed by kerosene. Given that the infrared energy from the target is very low, thermal isolation is important for reducing heat dissipation and enhancing device sensitivity. Owing to the low thermal conductivity of kerosene (0.2 W/m · K), immersing the MS in kerosene greatly increases the heat absorbed by kerosene. However, the 200 μm-thick supporting substrate of the MS has a much larger heat capacity than that of the MIM sandwich structure, whose thickness is less than 0.5 µm. As a result, a considerable amount of heat generated will be absorbed by the supporting substrate of the MS instead of kerosene. Therefore, a thermal isolation layer with superb thermal resistance and ultrasmall heat capacity is desired (the thermal isolation layer itself absorbs a negligible amount of heat).

Two-dimensional material heterostructures have extremely high thermal resistance in the vertical direction because the thickness of the monolayer 2D material is smaller than the mean free path of molecules, which results in strong phonon scattering at the interface^31,32^. The phonon density of states (PDOS) can describe the heat transfer effect at the nanoscale^33^. According to Vaziri et al.^34^:

3 $${TBC}\propto {Trans}.\times {PDOS\,overlap}\times {df}/{dT}$$

where *TBC* is the thermal boundary conductivity (a smaller *TBC* indicates a stronger thermal isolation capability) and *Trans* is the mass density mismatch. The *PDOS overlap* is the overlap between the PDOSs of two contacting materials, and *df/dT* is the derivative of the Bose‒Einstein distribution function with respect to temperature. The mass density mismatch between graphene and MoS_2_ is 0.448, and the number of modes for the PDOS overlap is 3.9 × 10^15^, indicating a low *TBC* and good thermal isolation properties of the 2D material heterostructure (graphene/MoS_2_)^34^. Additionally, atomically thin 2D material heterostructures have ultrasmall heat capacities. However, previous research focused on the thermal isolation characteristics of mechanically exfoliated micrometer-sized 2D material flakes, which are unsuitable for practical use. The thermal isolation characteristics of large-area CVD 2D material heterostructures remain unknown.

Here, we measured the thermal resistance of a large-area (graphene/MoS_2_/graphene) 2D material heterostructure and demonstrated its practical application in MEMS devices for the first time. Graphene and MoS_2_ are the most intensively studied 2D materials. Compared with other 2D materials, large-area graphene and MoS_2_ films synthesized by CVD have better properties, and the CVD technique for graphene/MoS_2_ growth is relatively reliable. In addition, graphene and MoS_2_ demonstrate good stability, whereas some 2D materials, such as black phosphorus, are unstable in ambient environments. Therefore, graphene and MoS_2_ were chosen for constructing vertical heterostructures. To measure the thermal resistance of the CVD graphene/MoS_2_/graphene heterostructure, the thickness of which is less than 2 nm, the heterostructure was transferred to the bottom side of the glass substrate, as shown in Fig. 4a, and a mechanically exfoliated small graphene flake was transferred to the top surface of the glass substrate as a microthermometer. We heated the bottom side of the glass substrate, and the temperature variation of the top graphene flake Δ*T*_*graphene*_ was measured via Raman microscopy as the graphene G peak shifted to −0.02 cm^−1^/K. We prepared samples without a 2D material heterostructure as the control group. Compared with the control samples, the samples with a heterostructure isolation layer clearly demonstrated a smaller Raman shift (Fig. 4c), indicating a smaller Δ*T*_*graphene*_ and superior thermal isolation characteristics of the CVD graphene/MoS_2_/graphene heterostructure. According to our experimental results, the larger-area CVD graphene/MoS_2_/graphene heterostructure has an ultralarge vertical direction thermal resistance of 4.2 K/W, and the effective thermal conductivity is 3.5 × 10^−6^ W/(m·K). The temperature distributions of the MS (with/without the graphene/MoS_2_/graphene heterostructure) in the infrared switch and surrounding kerosene after 20 s of infrared radiation were simulated via COMSOL (Fig. 4d). The graphene/MoS_2_/graphene heterostructure significantly reduces the heat flow from the MIM to the glass substrate. As a result, the kerosene above the MIM has a higher temperature (Fig. 4d). We then experimentally investigated the displacement of two-phase microfluidic flows heated by different MSs (infrared power intensity of 32 µW/mm^2^). With the graphene/MoS_2_/graphene heterostructure, the displacement of the microfluidic flow reached ~3000 µm after 30 s of infrared radiation, whereas the value greatly decreased to ~1500 µm without the graphene/MoS_2_/graphene heterostructure (Fig. 4e). Therefore, the 2D material heterostructure thermal isolation layer significantly enhances the device sensitivity.

### Sensing performance of the infrared switch

**Fig. 5:**
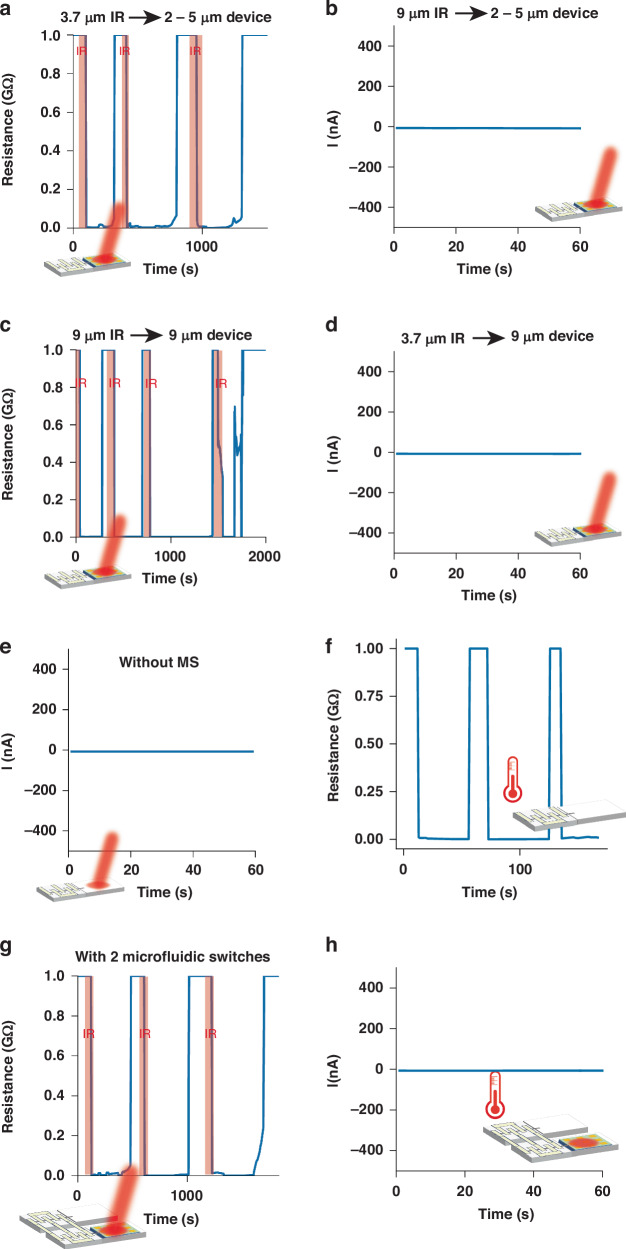
Sensing performance of the infrared switch. **a** Single microfluidic switch with the MS (absorption wavelength of 2–5 μm) under 3.7 μm infrared irradiation. **b** Single microfluidic switch with the MS (absorption wavelength of 2–5 μm) under 9 μm infrared irradiation. **c** Single microfluidic switch with the MS (absorption wavelength of 9 μm) under 9 μm infrared irradiation. **d** Single microfluidic switch with the MS (absorption wavelength of 9 μm) under 3.7 μm infrared irradiation. **e** Single microfluidic switch without the MS under infrared irradiation. **f** Single microfluidic switch under ambient temperature variation. **g** Infrared switch with a pair of microfluidic flows under infrared irradiation. **h** Infrared switch with a pair of two-phase microfluidics under ambient temperature variation

Figure 5a, b displays the characteristics of a two-phase microfluidic switch integrated with the MS whose absorption wavelength is 2–5 µm. Under 3.7 µm wavelength infrared irradiation, the microfluidic switch was turned on within ~15 s and slowly switched to the ‘off’ state as the infrared source was turned off. Figure 5a shows the excellent repeatability of our microfluidic switch. Notably, the upper limit of the digital multimeter is 10^9^ Ω; thus, the actual ‘off’ state resistance of our microfluidic switch exceeds 10^9^ Ω, and the on/off ratio can reach 10^6^. The same device showed no response to 9 µm wavelength infrared radiation (Fig. 5b), demonstrating excellent frequency selectivity. Figure 5c, d illustrates the characteristics of a two-phase microfluidic switch integrated with the MS whose absorption wavelength is 9 µm; it can only be activated by 9 µm infrared irradiation and showed no response to 3.7 µm infrared irradiation. The sensitivity of the switch is 257 µm/mW (1 mW infrared energy results in 257 µm displacement of the two-phase flow). In the control group, a two-phase microfluidic switch without the MS showed no response to (3.7 µm or 9 µm) infrared irradiation (Fig. 5e). Additionally, a single two-phase microfluidic switch was sensitive to environmental temperature changes (it turned on as the temperature increased to ~0.5 °C, Fig. 5f).

To eliminate the impact of environmental temperature variation, we fabricated a device with a pair of symmetric two-phase microfluidic switches, and only one microfluidic switch was integrated with the MS (absorption wavelength of 2–5 µm) in the reservoir. As demonstrated in Fig. 5g, h, the infrared switch was sensitive to (3.7 µm wavelength) infrared irradiation. It turned on within 20 s and maintained the ‘on’ state for ~300 s after the infrared source was turned off, demonstrating excellent switching performance and self-holding ability (Fig. 5g). Figure 5h shows that the infrared switch remained in the ‘off’ state without infrared irradiation as the ambient temperature changed (~0.5 °C), indicating that spurious triggering resulting from environmental temperature changes can be avoided.

### Near-zero-standby-power WSNs

**Fig. 6:**
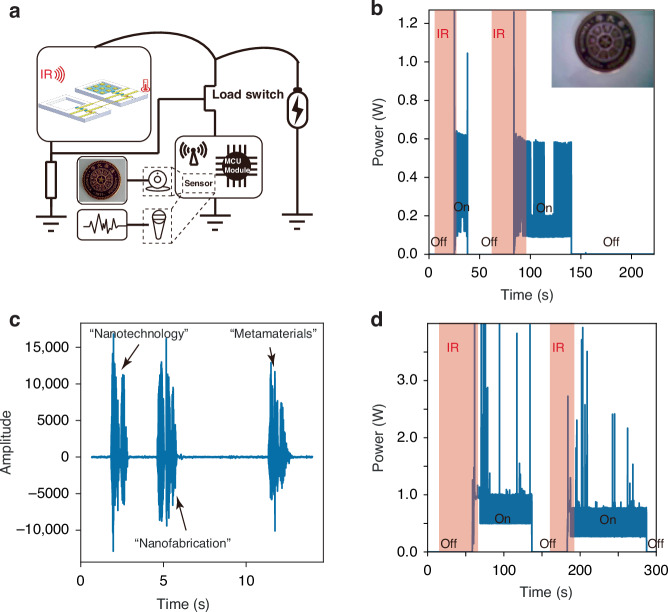
Near-zero-standby-power wireless sensor nodes. **a** Schematic of a WSN with an infrared switch, sensors, and a wireless communication module. **b** Power consumption of the WSN with a CCD and a wireless communication module. The WSN demonstrates near-zero standby power. The inset is a captured image received by a remote terminal (the logo of Tsinghua University). **c** Sensing performance of the WSN with a MEMS microphone. The audio waveform is received by a remote terminal, as the tester says “nanotechnology”, “nanofabrication”, and “metamaterials”. **d** Power consumption of the WSN with a MEMS microphone and a wireless communication module. The WSN demonstrates near-zero standby power

We realized near-zero-standby-power WSNs with visual/auditory functions by integrating the infrared switch, sensors (CCD/MEMS microphone), and wireless communication module (Fig. 6a). In the absence of infrared signals, the infrared switch was in the ‘off’ state. The WSNs were completely turned off and demonstrated near-zero standby power, as shown in Fig. 6b, d. As the infrared switch was illuminated by the target infrared signal, it turned on and activated the sensors integrated in the WSNs. In WSNs with visual function, the CCD captures an image (the logo of Tsinghua University) and transfers it to a remote terminal via a wireless communication module. The image received is shown in the inset of Fig. 6b. In WSNs with auditory function, a MEMS microphone was activated and started to record environmental sounds and transmit the information to a remote terminal. Figure 6c shows the audio waveform received as the tester said “nanotechnology”, “nanofabrication”, and “metamaterials”. Therefore, unattended WSNs with near-zero standby power were realized.

## Conclusion

In summary, we realized zero-power infrared switches by integrating an MS and two-phase microfluidic switches. The MS can recognize the infrared signals from the target and convert them into heat, which turns on the two-phase microfluidic switch. The impacts of the channel width, flow rate, and surface wettability on the properties of two-phase microfluidics were systematically investigated, and reliable two-phase microfluidic switches were realized on the basis of this fundamental study. The graphene/MoS_2_/graphene heterostructure has exceptional thermal isolation capability because strong phonon scattering reduces the heat flow from the MS to the substrate and significantly increases the device sensitivity. We realized near-zero-standby-power WSNs by integrating the infrared switches with a CCD/MEMS microphone and a wireless communication module. This work not only provides an approach for greatly lengthening the lifespan of WSNs but also may stimulate fundamental research and the application of two-phase microfluidics and nanoscale thermal isolation.
